# Paucicellular Fibroma of the Ascending Aorta

**DOI:** 10.1055/s-0041-1730006

**Published:** 2021-10-12

**Authors:** Stevan S. Pupovac, Elana Koss, James Albanese, Shahryar G. Saba, Alan R. Hartman, Robert Palazzo

**Affiliations:** 1 Department of Cardiovascular and Thoracic Surgery, Zucker School of Medicine at Hofstra/Northwell, Manhasset, New York; 2 Department of Cardiology, Zucker School of Medicine at Hofstra/Northwell, Manhasset, New York; 3 Department of Radiology, Zucker School of Medicine at Hofstra/Northwell, Manhasset, New York

**Keywords:** aorta, aortic root, aortic tumors, ascending aorta, cardiac tumors

## Abstract

Primary tumors of the aorta are extremely rare. To the best of our knowledge, herein, we present the first case in the literature of a paucicellular fibroma originating from the aortic wall.

## Introduction

Primary, intraluminal tumors of the aorta are exceedingly rare. Herein, we present the first case, to the best of our knowledge, in the literature of a paucicellular fibroma originating from the aortic wall.

## Case Presentation



**Video 1** Three-dimensional and two-dimensional transesophageal echocardiography, cardiovascular magnetic resonance imaging and intraoperative view of a paucicellular fibroma originating from the wall of the ascending aorta.


A 45-year-old female, who was being evaluated for primary hypertension, was found to have an abnormal nuclear stress test which prompted further evaluation. Coronary computed tomography (CT) angiography incidentally revealed a well-marginated, oval-shaped, homogenous, soft-tissue mass in the proximal ascending aorta, measuring 2.5 cm × 1.2 cm × 1.5 cm. The inferior aspect of this mass appeared contiguous with the anterior aspect of the aorta, immediately superior to the origin of the right coronary artery (RCA). Given its proximity to the RCA and for further assessment, transesophageal echocardiography (TEE) and cardiovascular magnetic resonance (CMR) imaging were performed.

TEE revealed similar findings to the CT images; an ovoid, mobile mass with an echolucent center located in the proximal ascending thoracic aorta that seemed to originate from the right sinotubular junction. Projecting into the proximal aortic lumen, it appeared attached to the wall of the aorta by a tiny stalk ( Video 1 ; available in the online version). As the mass did not clearly fit any known described lesions, the differential was broad and nonspecific; from an atypical papillary fibroelastoma to an atypical benign or malignant mass to an atypical thrombus.

CMR revealed a 1.8 cm × 1.2 cm mass originating in the proximal ascending aorta (at the level of the sinotubular junction), just superior to the right coronary sinus of Valsalva ( Video 1 ; available in the online version). The mass appeared adherent to the aortic wall, without significant independent motion. The mass appeared hyperintense on T1- and T2-weighted dark blood imaging. The mass did not demonstrate first pass-perfusion or late gadolinium enhancement. The differential diagnosis included thrombus and papillary fibroelastoma; however, the overall CMR findings were atypical for each of these.

**Fig. 1:**
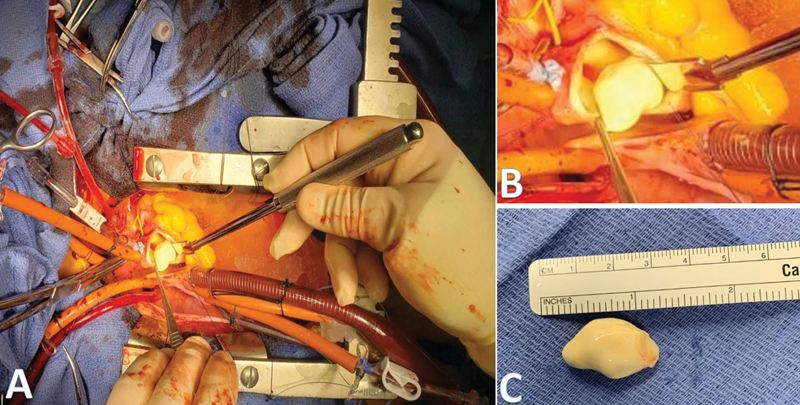
Intraoperative views of a paucicellular fibroma originating from the wall of the ascending aorta. ( **A** ) Panoramic view of the surgical field and mass emanating from the aortotomy of the ascending aorta. ( **B** ) Close-up view of the mass and opened ascending aorta. ( **C** ) Resected 2.3-cm paucicellular fibroma.

Given the unclear etiology and the potential for thromboembolic complications, the decision was made for surgical resection. A standard midline sternal incision was made, and cardiopulmonary bypass was established with cannulation of the proximal aortic arch and the right atrium. While cooling to 32°C, a left ventricular vent was placed via the right superior pulmonary vein. With the patient on vented bypass, the distal ascending aorta was cross-clamped and one dose of the del Nido cardioplegia was given. The aorta was opened and upon inspection, the mass appeared to be completely endothelialized. It was shaved off the aortic intima without any visible damage to the intima. A full-thickness resection was not ideal, as the mass originated at the ostium of the RCA, and would have required resecting part of the coronary artery ( Fig. 1 ). The mass was sent for frozen section evaluation which revealed a benign lesion. The coronary ostium and aortic valve were inspected and found unharmed, and the aorta was then closed in two layers with 4–0 prolene suture. The patient had an uneventful recovery and was discharged home on postoperative day 4.

**Fig. 2:**
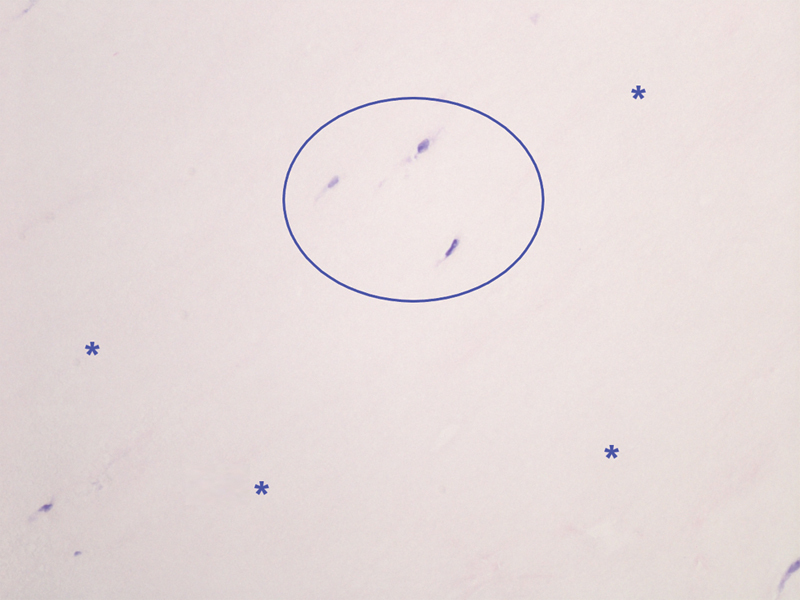
Histological examination (hematoxylin and eosin stain, original magnification ×200) of a paucicellular fibroma demonstrating gross acellularity and several fibroblasts (encircled by purple oval) amidst a hyalinized collagenous background (depicted by purple asterisks).

Final in-house pathology revealed an aortic mass with paucicellular fibrous tissue. Given then unusual findings, the specimen was sent for further evaluation to an outside institution. The mass was characterized as a benign, well-circumscribed, paucicellular collagenous tumor. Given the striking acellularity, extensive collagen, and mild amount of elastic fibers, the diagnosis of a paucicellular fibroma was made ( Fig. 2 ).

## Discussion

The first documented case of an aortic tumor was reported by Brodowski 1 in 1873. Since then, the publication of primary tumors of the ascending aorta has been limited to case reports. While both malignant (fibrous histiocytoma 2 ) and benign (fibroma 3 ) lesions have been described, benign fibroelastomas have been reported with the greatest frequency. 4

Given the rarity, varying histology, and mixed clinical manifestations of these tumors, preoperative or antemortem diagnosis is typically precluded. Cardiac fibromas primarily affect children and typically originate from the left ventricle, right ventricle, and interventricular septum. 5 Rarely, they have been reported to originate intraluminally, in the pulmonary artery, and ascending aorta. By comparison, greater than 80% of papillary fibroelastomas originate on valvular surfaces, with the aortic valve being the most common. 6 Extravalvular surfaces of the heart comprise the remaining 20%, with the following locations listed from most to least frequent: left ventricle, left atrium, left atrial appendage, atrial septum, right atrium, right atrial appendage, Eustachian valve, and right ventricle. 6

Grossly, cardiac fibromas appear as solid, firm, lesions that differ greatly in morphologically from papillary fibroelastomas which have been likened to sea anemones. Papillary fibroelastomas emanate from a stalked central core with projectile, frond-like arms. 6 7 Histologically, papillary fibroelastomas consist of avascular papillary tissue (avascular collagen and variable elastic tissue, surrounded by acid mucopolysaccharide) with a surrounding layer of endothelium. In fibromas, fibroblasts have been observed in a collagenous background, with varying numbers of elastic fibers. 6 7 Paucicellular fibromas are related to fibromas in gross appearance and histology, although are defined by their extreme acellularity. 6 7 8 While exceedingly rare, they have been depicted as cutaneous and tendon sheath lesions. To the best of our knowledge, this is the only case of a paucicellular fibroma originating from the aortic wall that has been reported in the literature.

Symptoms associated with aortic tumors are variable. While the majority of the small number of patients in literature were asymptomatic, extrapolating data from ventricular and valvular tumors suggest the possibility of serious complications including cerebrovascular accidents, myocardial infarction, or sudden death. 1 4 6

CMR provides the closest assessment of noninvasive histology compared with the other imaging modalities given its ability to characterize tissue. One important exception is calcified tissue which is best identified by CT.

Given the rarity of aortic tumors, therapeutic management is unclear and typically individualized. Resection of benign tumors, if technically feasible, may be advised given the potential for thromboembolic complications. Local recurrence is unlikely for benign lesions; however, given the lack of literature, follow-up should be tailored on a case-by-case basis. Our preference is repeat CT imaging at 1 year and then on an as-needed basis. The surgeon should be prepared for extensive reconstruction should the tumors involve the aortic valve, coronary ostia or aortic root.

In conclusion, to the authors' best knowledge, our report describes the first case of a paucicellular fibroma originating from the aortic wall. While tumors of the ascending aorta are rare, surgeons should be prepared for extensive reconstruction given the surrounding anatomy.
